# Contribution of Thromboxane A_2_ in Rat Common Carotid Artery Response to Serotonin

**DOI:** 10.3797/scipharm.1004-04

**Published:** 2010-06-15

**Authors:** Miroslav Radenković, Marko Stojanović, Mirko Topalović

**Affiliations:** Department of Pharmacology, Clinical Pharmacology and Toxicology; Medical Faculty; University of Belgrade; PO Box 38; 11129 Belgrade; Serbia

**Keywords:** Serotonin, Common Carotid Artery, Endothelium, Thromboxane A_2_

## Abstract

Serotonin is a vasoactive substance that in different blood vessels mostly induces vasoconstriction. Considering the important role of common carotid artery in brain blood supply, the aims of this study were to investigate the effect of serotonin on isolated rat common carotid artery and also to examine participation of intact endothelium, cyclooxygenase products, Ca^++^ channels and 5-HT_2_ receptors in serotonin-evoked action. Endothelium was mechanically removed from some vascular rings. Circular artery segments were placed in organ baths containing Krebs–Ringer bicarbonate solution. Cumulative concentration-contraction curves for serotonin were obtained in rings previously equilibrated at basal tone. Serotonin produced concentration-dependent contraction, which was unaltered by endothelial denudation. Serotonin-induced effect was notably and comparably reduced by indomethacin (cyclooxygenase inhibitor) or OKY–046 (thromboxane A_2_-synthase inhibitor) on intact or denuded rings. Nifedipine (Ca^++^ channel blocker) or ketanserin (5-HT_2_ receptor antagonist) strongly reduced serotonin-evoked effect. Our results suggest that serotonin produced concentration-dependent and endothelium-independent contraction of carotid artery, which was initiated by activation of 5-HT_2_ receptors located on smooth muscle cells and mediated via L-type Ca^++^ channels. Thromboxane A_2_ from smooth muscle cells notably contributed to the overall contraction of carotid artery induced by serotonin.

## Introduction

Serotonin is a monoamine neurotransmitter and vasoactive substance with well described vascular actions. In the vascular system serotonin predominantly induces endothelium-independent vasoconstrictions after binding to serotonin 5-HT_1_ and 5-HT_2_ receptors that are located on the smooth muscle cells. This is mainly described in the blood vessels of splanchnic region, kidneys, lungs and brain [1–4]. Moreover, serotonin may also additionally increase vascular contractions induced by noradrenaline, angiotensin II or thromboxane [5, 6]. Contrary to previous findings, it has been established that in different blood vessels serotonin induces vasoconstrictions that may be limited by protective function of intact endothelium through the release of nitric oxide or prostacyclin, but also, to a lesser extent, via inhibition of noradrenaline release from perivascular sympathetic neurons [7–9]. In addition, it has been unevenly described that other vasoactive autacoids can contribute to serotonin–induced vascular contractions [10–12].

Common carotid artery has a crucial role in the brain blood supply. On the other hand, it has been established that intimal thickening, which is usually associated with endothelial dysfunction and advancement of atherosclerosis, may increase reactivity of this blood vessel to serotonin [13]. This has a significant clinical importance considering that positive correlation between thickenings of intimal or muscular layer of carotid artery and subsequent cerebrovascular events has been established [14]. Furthermore, carotid atherosclerosis has been found to be associated with the extent and severity of coronary artery disease [15]. In regard to serotonin action, it was also described that the constrictor response of the rabbit carotid artery to this monoamine was increased in diabetic animals [16].

Taking into consideration that serotonin actions on the isolated carotid artery from different species are still under investigation, the present experiments were undertaken in order:

1. to examine the effect of serotonin on the isolated rat common carotid artery;
2. to investigate possible participation of intact endothelium and cyclooxygenase products in serotonin-evoked action;
3. to establish if Ca^++^ channels are important for transduction mechanism related to serotonin effect, and
4. to determine if serotonin 5-HT_2_ receptors are involved in serotonin-produced response of the investigated blood vessel.

## Materials and Methods

The animal procedures were in full accordance with the standards from the European Convention for the Protection of Vertebrate Animals Used for Experimental and other Scientific Purposes, as well with guidelines from the Good Laboratory Practice. The left and the right common carotid arteries were isolated from male Wistar rats weighing 220–280 g. Blood vessels were carefully dissected from the surrounding fat and connective tissue, further cut into circular segments (which were 4 mm long) and immediately placed in Krebs-Ringer bicarbonate solution. The endothelium was removed from some rings by gently rubbing the intimal surface with stainless-steel wire. Ring preparations were mounted between two stainless-steel triangles in an organ bath containing 15 ml Krebs-Ringer bicarbonate solution (37°C, pH 7.4), aerated with 95% O_2_ and 5% CO_2_. One of the triangles was attached to a displacement unit allowing a fine adjustment of tension, and further connected to a force-displacement transducer (Hugo Sachs Elektronik F30 Type 372, Freiburg, Germany). Isometric tension was continuously recorded on a Rikadenki R-62 multi-pen electronic recorder (Rikadenki Kogyo CO., LTD, Tokyo, Japan). The preparations were allowed to equilibrate for 45 min in Krebs-Ringer bicarbonate solution. During next 30 min each vascular ring was gradually stretched to the resting tension of 1.5 g, and additionally equilibrated for 30 min before experimentation.

**Fig. 1.**
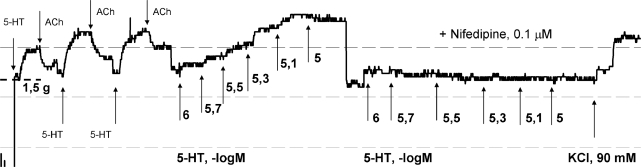
A sample recording representing the inhibitory effect of nifedipine on serotonin-induced contraction on isolated rat common carotid artery.

At the beginning of each experiment endothelial functional integrity was examined by precontraction of isolated common carotid artery with submaximal concentration (EC_50_–EC_70_) of serotonin (1–10 μM), followed by the addition of acetylcholine (1 μM). This procedure was repeated three times in 20 min intervals (Fig. 1). Relaxation >80% of initial serotonin contraction was indicative for structurally intact endothelium.

Cumulative concentration-contraction experimental curves for 5-HT (1–10 μM) were obtained in rings previously equilibrated at basal tone. The higher concentration of agonist was administered in an organ bath only after the equilibrium response of the lower concentration was produced (Fig. 1). Since preliminary experiments in preparations of carotid artery demonstrated that the first and the second concentration-contraction curve (determined 45 min apart) for 5-HT were not significantly different, a multiple curve design protocol was applied. Therefore, a pharmacological blocker of interest was incubated between two concentration-contraction curves for 5-HT during the period of 30 min. At the end of each experiment vascular rings were additionally equilibrated for 30 min at the level of resting tension and finally contracted with K^+^-rich Krebs-Ringer-bicarbonate solution prepared by equimolar replacement of 90 mM NaCl with 90 mM KCl.

The contraction induced by each concentration of 5-HT is expressed as a percentage of the contraction produced by 90 mM KCl (100%), and it was used in the construction of the concentration–response curves. The concentration of 5-HT producing 50% of maximum response (EC_50_) was determined for each curve, and finally presented as pD_2_ (pD_2_ = –log EC_50_). The results are expressed as means ± S.E.M.; n refers to the number of experiments. Statistical significance of differences between two means was determined with Student’s *t*-test for paired or unpaired observations were appropriate. A value of p<0.05 was considered to be statistically significant.

The Krebs–Ringer bicarbonate solution had the following composition (in mM): NaCl 118.3; KCl 4.7; CaCl_2_ 2.5; MgSO_4_ 1.2; KH2PO_4_ 1.2; NaHCO_3_ 25.0; Ca-EDTA 0.026; glucose 11.1. The following drugs were used: indomethacin (Sigma, St Louis, USA); acetylcholine iodide, (Serva, Heidelberg, Germany), 5-HT, ketanserin, nifedipine (ICN, Irvine, CA, USA) and (*E*)-3-(4-(1-imidazolylmethyl)phenyl)-2-propenoic acid) – OKY-046 (Ono Pharmaceutical, Osaka, Japan). All agents were dissolved in distilled water (except as described below) and diluted to the desired concentration with buffer. Stock solutions (100 μM) of indomethacin and nifedipine were prepared in equimolar Na_2_CO_3_ solution and 70% ethanol, respectively. Preliminary experiments in preparations of rat carotid artery demonstrated that the vascular action of 5-HT was unaffected by final concentration of used dissolvents. Likewise, the basal tone of carotid artery remained unaltered during 30 min treatment with pharmacological blockers. The experiments with nifedipine were performed in a dark room. During experimental procedure all agents were added directly to the bath in a volume of 0.15 ml and the concentrations given are the calculated final concentrations in the bath solution.

## Results

**Fig. 2.**
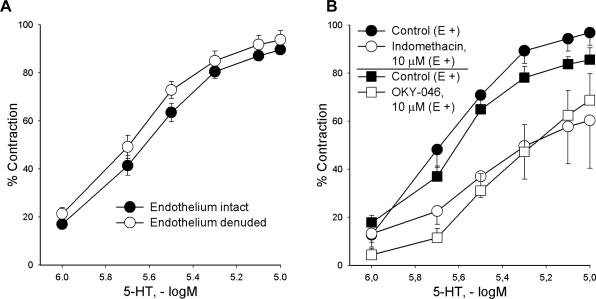
The effects of endothelium removal (A) or indomethacin and OKY– 046 (B) on serotonin–produced vascular response of the common carotid artery. Each point represents mean ± S.E.M. (n ≥ 4). Contractile responses are expressed as percentages of contraction induced by Krebs-Ringer bicarbonate solution with 90 mM KCl. E(+) = endothelium intact.

Serotonin (1–10 μM) produced concentration-dependent contraction of isolated rat carotid artery (pD_2_ = 5,66 ± 0,01; max. contraction = 89,64 ± 2,01 %; Fig. 2A). Endothelial denudation did not change control contractions of serotonin (pD_2_ = 5,72 ± 0,01; max. contraction = 93,75 ± 3,78 %; p>0,05 *vs.* control; Fig 2A).

Vascular response of intact rings to serotonin was notably reduced by 10 μM indomethacin – a cyclooxygenase inhibitor (control: pD_2_ = 5,68 ± 0,01; max. contraction = 96,89 ± 5,24 %; indomethacin: pD_2_ = 5,52 ± 0,06; max. contraction = 60,33 ± 20,00 %; p<0,05 *vs.* control; Fig. 2B). Comparable inhibition of serotonin-evoked effect was also obtained in vascular preparations without endothelium (control: pD_2_ = 5,79 ± 0,01; max. contraction = 87,26 ± 3,04 %; indomethacin: pD_2_ = 5,59 ± 0,02; max. contraction = 49,69 ± 4,03 %; p<0,01 *vs.* control; Fig. 2B).

OKY-046 (a thromboxane A_2_-synthase inhibitor, 10 μM) reduced serotonin-induced contractions on intact vascular rings in a similar extent comparing to indomethacin (control: pD_2_ = 5,67 ± 0,03; max. contraction = 85,59 ± 4,94 %; OKY-046: pD_2_ = 5,40 ± 0,04; max. contraction = 68,75 ± 10,96 %; p<0,05 *vs.* control). Equivalent reduction of serotonin-induced action was also obtained in vascular preparations without endothelium (control: pD_2_ = 5,68 ± 0,04; max. contraction = 91,30 ± 3,21 %; OKY-046: pD_2_ = 5,29 ± 0,10; max. contraction = 57,02 ± 11,67 %; p<0,05 *vs.* control).

**Fig. 3.**
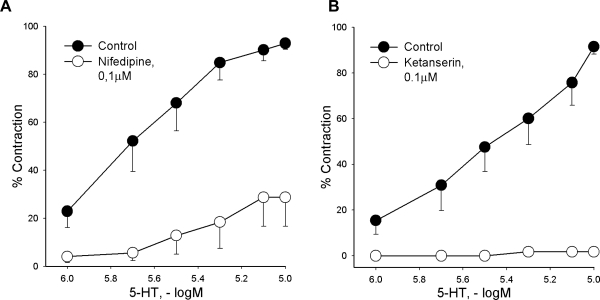
The effect of nifedipine (A) or ketanserin (B) on serotonin-produced vascular response of the common carotid artery. Each point represents mean ± S.E.M. (n ≥ 4). Contractile responses are expressed as percentages of contraction induced by Krebs-Ringer bicarbonate solution with 90 mM KCl.

Nifedipine (a voltage-gated L-type Ca^++^ channel blocker, 0.1 μM) strongly inhibited vascular response of intact carotid artery to serotonin (control: max. contraction = 92,94 ± 2,50 %; nifedipine: max. contraction = 28,75 ± 12,07 %; p<0,01 *vs.* control; Fig. 3A). Furthermore, ketanserin (5-HT_2_ receptor antagonist, 0.1 μM) abolished serotonin – evoked contraction of examined blood vessel (control: max. contraction = 91,53 ± 3,27 %; ketanserin: max. contraction = 1,79 ± 1,09 %; p<0,01 *vs.* control; Fig. 3B).

## Discussion

Serotonin is endogenous monoamine that is involved in regulation and control of many physiological functions, including vascular resistance of different blood vessels. Taking into account previous studies considering mechanisms involved in action of serotonin on various vascular beds, the present experiments were undertaken in order to examine the effect of serotonin in the isolated rat common carotid artery. The results of our experiments have shown that serotonin produced concentration-dependent contraction of the carotid artery, which was unaffected by endothelial denudation. Consequently, it can be proposed that the contractile response of examined blood vessel to serotonin does not depend upon functional integrity of endothelial layer. Accordingly, maximal serotonin-evoked contraction of the carotid artery also did not change after endothelial removal in normotensive Wistar-Kyoto rats [17], although in this investigation the authors have obtained higher pD_2_ value (6,00) than we reported here in our study (5,72).

It has been previously reported that in the rat femoral artery contractions induced by serotonin were attenuated by cyclooxygenase inhibition, thus suggesting that prostaglandin production may contribute to serotonin-induced action [18]. The results from another investigation on the rabbit carotid artery further implicated a vasoconstrictor prostanoid of endothelial origin to be important for serotonin-evoked effect [16]. In our experiments indomethacin reduced contractile response of the rat carotid artery to serotonin in comparable extent on rings with and without endothelium. The equivalent result was obtained after inhibition of thromboxane A_2_-synthase. Based on these findings it can be suggested that the rat carotid artery response to serotonin notably depends upon the production of cyclooxygenase vasoconstrictor metabolite, most probably thromboxane A_2_. According to our observations it also appears that thromboxane A2 is most likely released from the smooth muscle cells. This is also indirectly in accordance with the result that in the endothelium-denuded preparations of porcine coronary artery, a prostanoid TP receptor agonist (U-46619) augmented serotonin-induced contraction [19].

A major physiological event in the vascular smooth muscle cell contraction is an increase in intracellular calcium concentration via either the release from intracellular stores and/or from influx from the extracellular space [20]. The contribution of extracellular calcium on the carotid artery response to serotonin was evaluated by pretreatment of vascular preparations with nifedipine, a voltage-gated L-type Ca^2+^ channel blocker. Nifedipine strongly inhibited serotonin-evoked contraction, which is indicative for the assumption that the activation of L-type Ca^2+^ channels and subsequent influx of calcium ions extensively contributes to the overall transduction mechanism included in serotonin action on the rat carotid artery.

Vascular effects of serotonin are predominantly induced after initial binding of this monoamine to 5-HT_1_ or/and 5-HT_2_ receptors. Still, in previous investigations on the isolated rabbit carotid artery it was reported that only 5-HT_2_ receptors were involved in contractions induced by serotonin [21, 22]. Accordingly, in our investigation ketanserin (a 5-HT_2_ receptor antagonist) abolished contractile response of the rat carotid artery to serotonin. Taking into account that in our experiments serotonin produced endothelium-independent action, it can be assumed that the contractile effect of investigated monoamine largely depends upon activation of 5-HT_2_ receptors that are located on the smooth muscle cells.

In conclusion, our results suggest that serotonin produced concentration-dependent and endothelium-independent contraction of the rat carotid artery, which was initiated by activation of 5-HT_2_ receptors located on the smooth muscle cells and mediated via L-type Ca^++^ channels. In addition, it was found that thromboxane A_2_ from the smooth muscle cells notably contributed to the overall contraction of the carotid artery induced by serotonin.
